# A 3-Year Survival Update from a Phase 2 Study of Paclitaxel Plus Cisplatin and 5-Fuorouracil Induction Chemotherapy for Locally Advanced Borderline-Resectable Esophageal Squamous Cell Carcinoma: The NEOCRTEC-1601 Clinical Trial

**DOI:** 10.1245/s10434-023-14513-0

**Published:** 2023-11-02

**Authors:** Jia-Di Wu, Zhi-Qiang Wang, Qiao-Qiao Li, Zhi-Chao Li, Chao Ren, De-Shen Wang, Ji-Yang Chen, Qiong Tan, Yu-Hong Li, Hong Yang

**Affiliations:** 1 Department of Medical Oncology, State Key Laboratory of Oncology in South China, Sun Yat-sen University Cancer Center, Guangzhou, People’s Republic of China; 2 Department of Thoracic Surgery, State Key Laboratory of Oncology in South China, Sun Yat-sen University Cancer Center, Guangzhou, People’s Republic of China

## Abstract

**Background:**

This study updated 3-year analyses to further characterize the impact of docetaxel, cisplatin, and fluorouracil (TPF) chemotherapy followed by surgery.

**Methods:**

This study was a single-center phase 2 clinical trial. Patients with a diagnosis of borderline resectable esophageal squamous cell carcinoma (BR-ESCC) because of the primary tumor or bulky lymph node that potentially invaded adjacent organs were eligible. The treatment started with TPF chemotherapy followed by surgery if the cancer was resectable, or by concurrent chemoradiation if it was unresectable. This updated report presents the 3-year overall survival (OS) and progression-free survival (PFS) rates.

**Results:**

Surgery was performed for 27 patients (57.4%), and R0 resection was confirmed in 25 patients (53.2%). Pathologic complete response was confirmed in four patients (8.5%). The median follow-up time for the surviving patients was 44.8 months (range, 3.4–74.6 months). The median OS for all the patients was 41.9 months (95% confidence interval [CI], 18.6–65.3 months), with a median PFS of 38.7 months (95% CI, 23.5–53.9 months). The 3-year survival rate for all the patients was 54.4%. The 3-year survival rate for the R0 patients was 65.4%.

**Conclusion:**

Long-term follow-up evaluation confirmed that TPF followed by surgery is feasible and promising in terms of survival for BR-ESCC patients.

*Trial Registration* ClinicalTrials.gov identifer: NCT02976909.

Esophageal cancer is the ninth most frequent cancer and the sixth leading cause of cancer-associated death in the world.^1^ More than 90% of esophageal cancer patients in East Asia have esophageal squamous cell carcinoma (ESCC).^2^ Currently, the standard treatment for locally advanced resectable ESCC is neoadjuvant chemoradiotherapy (NCRT) or neoadjuvant chemotherapy (NCT) followed by surgery, and definitive chemoradiotherapy (DCRT) without planned esophagectomy is considered the standard treatment for patients with unresectable ESCC.^3–6^

In general, there is a high risk of esophageal carcinoma invading adjacent organs and structures such as the aorta, arch vessels, airways, vertebral body, and pericardium due to lack of serosa in the esophagus. Suspicious organ involvement occurs in some patients with clinical stage T4 and/or bulky lymphadenopathy, which cannot be definitively diagnosed as T4b. These patients are considered to have borderline resectable esophageal squamous cell carcinoma (BR-ESCC), and may have the opportunity to undergo R0 resection.^7, 8^

We performed a phase 2 trial^8^ (NCT02976909/NEOCRTEC-1601) to evaluate the efficacy and safety of paclitaxel in combination with cisplatin and 5-fluorouracil (TPF) induction chemotherapy for BR-ESCC. The clinical trial included 47 patients with BR-ESCC from Sun Yat-sen University Cancer Center from July 2014 to February 2019. The results showed that 27 patients (57.4%) received surgery, and the R0 resection rate as the primary end point was 53.2%. Pathologic complete response was confirmed in four patients (8.5%).

After a median follow-up period of 16 months, the results showed significant benefits in both median overall survival (OS) and progression-free survival (PFS) favoring the surgery patients (median OS, 33.3 vs 14.1 months ([hazard ratio [HR], 0.32; 95 confidence interval [CI], 0.12–0.88; *p* = 0.027); median PFS,. 20.4 vs 9.9 months (HR, 0.426; 95% CI, 0.186–0.973; *p* = 0.032). Furthermore, TPF induction chemotherapy and conversion surgery were tolerable.

The current study aimed to compare treatment efficacy between R0 resection and non-R0 resection for patients with a diagnosis of BR-ESCC. Updated outcomes are reported to provide insight into the durability of the efficacy of TPF chemotherapy plus surgery after a longer follow-up period.

## Patients and Methods

The study was approved by the ethics committee of Sun Yat-sen University Cancer Center. All patients included in the study provided written informed consent before enrollment. The study was registered at ClinicalTrials.gov (NCT02976909).

### Patients

As previously reported,^8^ the study enrolled patients with histologically proven BR-ESCC. The patients eligible for enrollment were 18–70 years old, had an Eastern Cooperative Oncology Group (ECOG) performance status score of 0–1, and had adequate hematologic, hepatic, and renal function. Patients were excluded if they had cervical tumor, distant metastasis, history of other cancer, previous anti-tumor treatment, serious complications, active infection, severe hypersensitivity to chemotherapy drugs, or psychiatric illness.

### Study Design and End Points

The NEOCRTEC-1601 study was a single-center phase 2 clinical trial. The details of the borderline resectable criteria and clinical staging were described in our previous report.^8^ The primary end point was the R0 resection rate. The secondary end points were OS, PFS, adverse events, postoperative complications, and pathologic response. Overall survival was defined as the time from the registration date to the date of death or last follow-up visit. Progression-free surrival was defined as the time from the registration date to the date of disease progression or death.

### Treatment Procedure

The protocol therapy started with two or three cycles of TPF-induction chemotherapy (intravenous [IV] paclitaxel 135 mg/m^2^ on day 1; cisplatin 75 mg/m^2^ IV on day 1; 5-fluorouracil 4 g/m^2^ continuous infusion for 120 h from day 1 to day 5) every 3 weeks. The toxic effects of chemotherapy were assessed according to the National Cancer Institute’s Common Terminology Criteria for Adverse Events (CTCAE), version 5.0.

Afterward, if curative resection was considered possible on multidisciplinary consultation reassessment, the patient was scheduled for conversion surgery 3–4 weeks after induction therapy. McKeown esophagectomy combined with two-field lymphadenectomy was performed. The grade of postoperative complications was defined according to the Clavien-Dindo classification. In addition, chemoradiotherapy was recommended for patients receiving R1/R2 resection.

Concurrent chemoradiotherapy was administered as the main treatment for the patients who had tumors still considered unresectable after reassessment. If no tumor progression occurred during induction chemotherapy, the paclitaxel plus cisplatin (TP) regimen was recommended.

The gross tumor volume (GTV) encompassed the primary tumor and enlarged regional lymph nodes, as determined by imaging and endoscopy examinations. The clinical target volume (CTV) was determined by a GTV plus a 0.8- to 1.0-cm lateral margin and a 3.0- to 5.0-cm craniocaudal margin that included subclinical involvement. The planning target volume was defined as a CTV plus a 0.5- to 0.8-cm margin. The total planned dose for the planning target volume was 56–60 Gy in 2-Gy fractions per day 5 days a week. All the patients were irradiated by external beam radiation using 6 to 8-MV photon energies of the intensity-modulated radiation technique.

### Pathologic Analysis

Evaluations of residual tumor (R) were classified as follows: R0 resection (no microscopic and macroscopic residual tumor), R1 resection (only microscopic residual tumor), and R2 resection (macroscopic residual tumor). A condition without grossly and microscopically viable tumor in the entire surgical specimen, including the primary site and any resected lymph nodes, was defined as a pathologic complete response (pCR).

The American Joint Committee on Cancer (AJCC)/College of American Pathologists (CAP) tumor regression grade (TRG) was classified as follows: TRG 0 (pCR: no residual tumor cells), TRG 1 (near complete response: single or small groups of residual tumor cells), TRG 2 (partial response: residual tumors with degeneration of tumor cells and desmoplastic response), TRG 3 (poor or no response: a large number of tumor cells observed with little or no response to treatment).^9^

### Statistical Analysis

According to the retrospective data from our center, the R0 resection rate for BR-ESCC with surgery alone was approximately 20% (3/12) and expected to increase to 40%. With a one-sided alpha significance level of 0.05 and a statistical power of 80%, the inclusion of 40 patients was required for evaluation of the R0 resection rate. In addition, assuming a dropout rate of 15%, at least 46 cases needed to be enrolled. The 95% CI for the R0 resection rate was calculated based on the binomial distribution.

Both OS and DFS were estimated using the Kaplan-Meier method, and a log-rank test was used for comparisons between the groups. In addition to comparing survival between the surgery and non-surgery patients, we divided the patients into two groups based on whether they underwent R0 resection. The patients in the R0 group included those who underwent R0 operation. The patients in non-R0 group comprised two types of patients: those who did not undergo surgery (*n *= 20) and those who received surgery without a successful R0 resection (*n *= 1).

The chi-square test or Fisher’s exact test and Student’s *t* test were used to analyze the differences in clinical features between the surgery and non-surgery patients. The 95% CI of the survival rates were calculated with the Clopper-Pearson method. Two-sided *p* values lower than 0.05 were considered statistically significant. All analyses were performed using R 4.0.1 (The R Project for Statistical Computing, Vienna, Austria) and IBM SPSS 24.0 (IBM, Chicago, IL, USA).

## Results

### Patients and Treatment

**Fig. 1:**
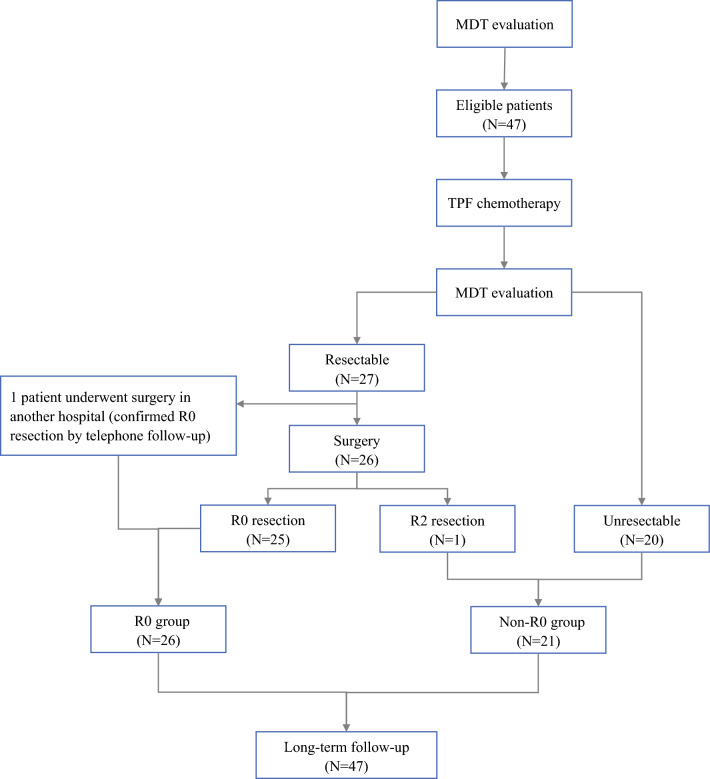
Consort flow diagram

Between July 2004 and February 2019, the study enrolled 47 BR-ESCC patients from Sun Yat-sen University Cancer Center. The accrual procedure, safety profiles, and treatment profiles were presented in our previous report.^8^ As treatment protocol, conversion surgery was performed for 27 patients (57.4%), including 1 patient who underwent conversion surgery at another hospital. After reassessment, 20 patients were considered unresectable, but only 11 patients received DCRT (Fig. 1).

For 25 of the 26 patients who underwent surgery at Sun Yat-sen University Cancer Center (53.2%; 95% CI, 38.9–67.5%), R0 resection was achieved, whereas 1 patient (2.1%) underwent R2 resection. In addition, we realized by telephone follow-up assessment that the patients who underwent surgery at another hospital also achieved R0 resection.

Table 1 shows the baseline characteristics of the 27 patients who underwent surgery and the remaining 20 patients who did not. Table 2 shows the clinical stage before and after induction treatment. There was no statistically significant difference between the surgery and non-surgery groups in age, sex, performance, status, tumor location, tumor differentiation, clinical *T* stage, or clinical *N* stage.

**Table 1:** Baseline patient characteristics

Characteristic	*n* (*n* = 47) *n* (%)	Surgery group (*n* = 27) *n* (%)	Non-surgery group (*n* = 20) *n* (%)	*p* Value
*Age (years)*				0.819
Median	60	61	60	
Range	45–70	45–70	46–69	
*Sex*				0.534
Male	38 (80.9)	21 (77.8)	17 (85.0)	
Female	9 (19.1)	6 (22.2)	3 (15.0)	
*Smoking history*				0.77
Yes	20 (42.6)	11 (40.7)	9 (45.0)	
No	27 (57.4)	16 (59.3)	11 (55.0)	
*BMI (kg/m*^*2*^*)*				0.397
< 24	40 (85.1)	24 (88.9)	16 (80.0)	
≥ 24	7 (14.9)	3 (11.1)	4 (20.0)	
*ECOG score*				0.905
0	31 (66.0)	18 (66.7)	13 (65.0)	
1	16 (34.0)	9 (33.3)	7 (35.0)	
*Main tumor location*				0.821
Upper	4 (8.5)	2 (7.4)	2 (10.0)	
Middle	29 (61.7)	17 (63.0)	12 (60.0)	
Lower	14 (29.8)	8 (29.6)	6 (30.0)	
*Tumor differentiation*				0.229
Well	2 (4.3)	2 (7.4)	0 (0)	
Moderate	32 (68.1)	16 (59.3)	16 (80.0)	
Poor	13 (27.7)	9 (33.3)	4 (20.0)	
*Cycles of completed chemotherapy*				0.322
1	2(4.3)	0(0)	2 (10.0)	
2	27 (57.4)	16 (59.3)	11 (55.0)	
3	17 (36.2)	10 (37.0)	7 (35.0)	
4	1 (2.1)	1 (3.7)	0 (0)	

*BMI*, body mass index; *ECOG*, Eastern Cooperative Oncology Group

**Table 2:** Clinical stage before and after chemotherapy

Characteristic	*n* (*n* = 47) *n* (%)	Surgery group (*n* = 27) *n* (%)	Non-surgery group (*n* = 20) *n* (%)	*p* Value
*Clinical T stage*				0.202
3	4 (8.5)	2 (7.4)	2 (10.0)	
4a	28 (59.6)	19 (70.4)	9 (45.0)	
4b	15 (31.9)	6 (22.2)	9 (45.0)	
*Clinical N stage*				0.330
0	2 (4.3)	0	2 (10.0)	
1	22 (46.8)	13 (48.1)	9 (45.0)	
2	15 (31.9)	10 (37.0)	5 (25.0)	
3	8 (17.0)	4 (14.8)	4 (20.0)	
*T stage after chemotherapy*				< 0.001
2	3 (6.4)	3 (11.1)	0	
3	25 (53.2)	22 (81.5)	3 (15.0)	
4a	12 (25.5)	1 (3.7)	11 (55.0)	
5	7 (14.9)	1 (3.7)	6 (20.0)	
*N stage after chemotherapy*				0.210
0	3 (6.4)	2 (7.4)	1 (5.0)	
1	26 (55.3)	18 (66.7)	8 (40.0)	
2	14 (29.8)	6 (22.2)	8 (40.0)	
3	4 (8.5)	1 (3.7)	3 (15.0)	

### Surgery and Postoperative Pathologic Results

McKeown surgery was performed for 24 of the 26 patients who received surgery at our hospital, whereas 1 patient underwent Sweet surgery and 1 patient underwent transperitoneal surgery due to their own will. For 25 patients (96.15%), R0 resection was performed, and 1 patient (2.1%) underwent R2 resection because the tumors clearly had invaded the left main bronchus. Pathologic complete response (pCR) was achieved in four patients (15.38%, 8.5% (*n *= 47). Other surgical details and pathologic results are shown in Table 3.

**Table 3:** Surgery and pathologic results

Datas	*n* = 26 *n* (%)
Mean operation time (min)	302.1
Median blood loss: ml (range)	100 (50–800)
Mean no. of LNDs	33.58
Mean no. of LND stations	10.77
Median hospital stay: days (IQR)	11.50 (9.00–17.50)
Cervical LND	4 (15.38)
*Evaluations of R*	
R0	25 (96.15)
R2	1 (3.85)
*Surgery options*	
Mckeown	24 (92.31)
Sweet	1 (3.85)
Peritoneal surgery	1 (3.85)
*Anastomotic location*	
Neck	24 (92.31)
Chest	1 (3.85)
Abdomen	1 (3.85)
*Substitution*	
Stomach	25 (96.15)
Colon	1 (3.85)
*Stage*	
I	7 (26.92)
pCR	4 (15.38)
Non-pCR	3 (11.54)
II	5 (19.23)
IIIA	1 (3.85)
IIIB	11 (42.31)
IVA	2 (7.69)

*LND*, lymph node dissection; *IQR*, interquartile range; *pCR*, pathologic complete response

### Survival

**Fig. 2:**
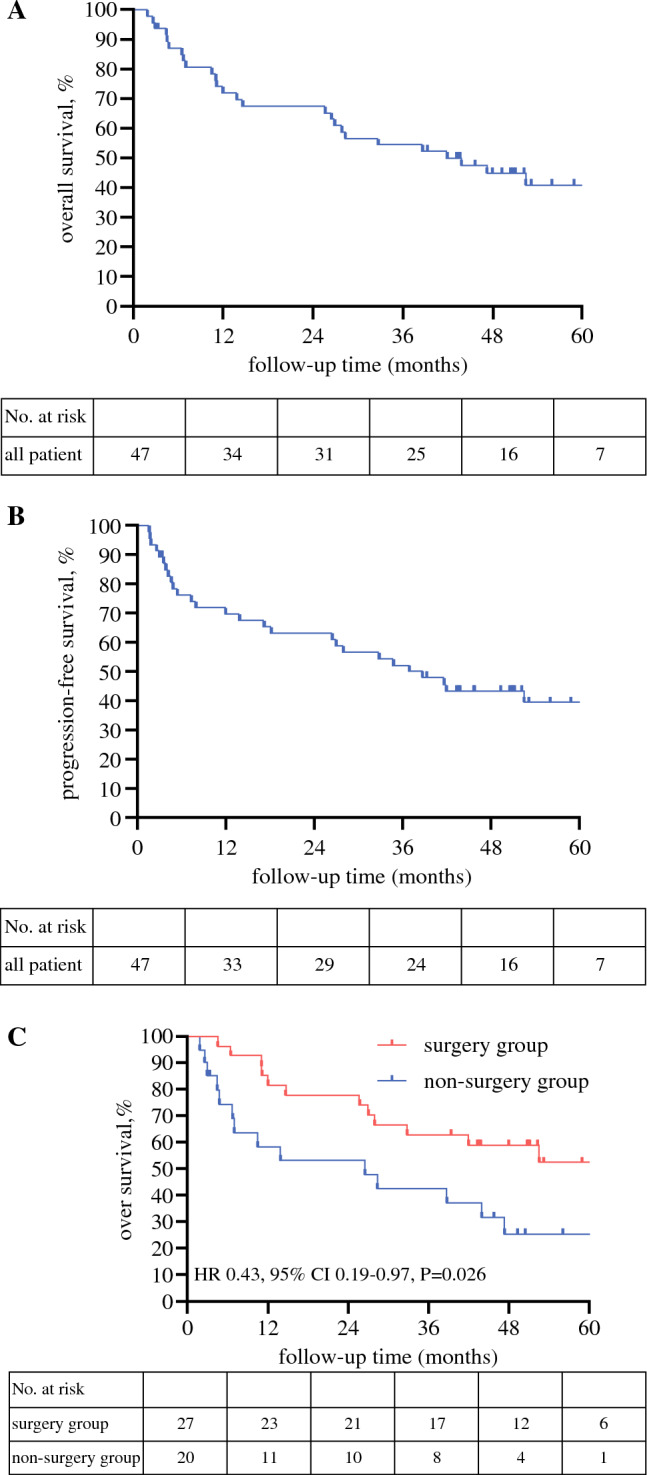

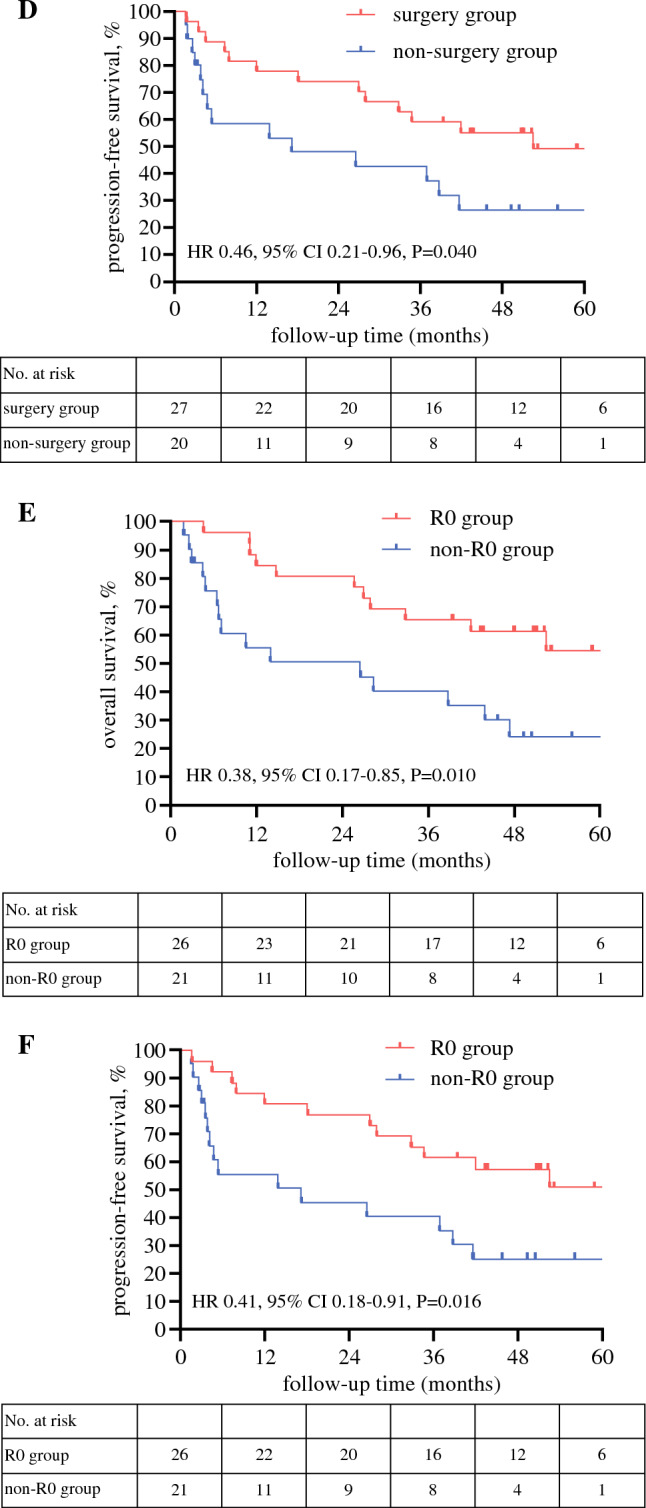
Overall survival and progression-free survival. **A** Overall survival of all patients. **B** Progression-free survival of all patients. **C** Overall survival of surgery and non- surgery groups. **D** Progression-free survival of surgery and non-surgery groups. **E** Overall survival of R0 and non-R0 resection groups. **F** Progression-free survival of R0 and non-R0 resection groups

The median follow-up time for the surviving patients was 44.8 months (range, 3.4–74.6 months). The median OS for all the patients was 41.9 months (95% CI, 18.6–65.3 months), and the median PFS for all the patients was 38.7 months (95% CI, 23.5–53.9 months). The 3-year survival rate was 54.4% (95% CI, 40.2–69.5 %), and the 3-year PFS rate was 52.3% (95% CI, 36.3–65.7%) (Fig. 2A and B).

As mentioned in our previous report, the patients were divided into two groups according to whether they underwent surgery. The non-surgery group comprised two types of patients: the patients who received DCRT without surgery and those who received neither DRCT nor surgery. The median OS was significantly more favorable in the surgery group than in the non-surgery group (not reached vs 26.5 months; HR, 0.43; 95% CI, 0.19–0.97); *p *= 0.026; Fig. 2C). Kaplan–Meier analysis for PFS also showed a significant difference between the two groups. The median PFS was 52.5 months in the surgery group compared with 17.2 months in the non-surgery group (HR, 0.46; 95% CI, 0.21–0.96; *p *= 0.040; Fig. 2D).

Furthermore, in accordance with whether they underwent R0 resection, the patients were divided into the R0 group (*n *= 26) and the non-R0 group (*n *= 21). The non-R0 group comprised two types of patients: those who did not undergo surgery (*n *= 20) and those who received surgery with unsuccessful R0 resection (*n *= 1).

The OS for the R0 group was significantly longer than for the non-R0 group (not reached vs 26.5 months; HR, 0.38; 95% CI, 0.17–0.85; *p *= 0.010; Fig. 2E). The 3-year survival rate was 65.4% (95% CI, 44.4–82.1%) for the R0 group compared with 40.3% (95% CI, 22.6–66.6%) for the non-R0 group (HR, 0.41; 95% CI, 0.16–0.91; *p *= 0.034). The median PFS was significantly more favorable in R0 group than in non-R0 group (not reached vs 17.2 months; HR, 0.41; 95% CI, 0.18–0.91; *p *= 0.016). The PFS rate at 3 years was 61.5% (95% CI, 40.7–79.1%) in the R0 group compared with 40.5% (95% CI, 22.6–65.6%) in the non-R0 group (HR, 0.46; 95% CI, 0.19–1.10; *p *= 0.059).

## Discussion

The NEOCRTEC-1601 trial was a prospective study investigating the efficacy of induction chemotherapy for BR-ESCC patients. To our knowledge, this phase 2 study was the first prospective clinical trial to demonstrate that TPF chemotherapy followed by surgery is an effective and safe treatment strategy.

The final results after long-term follow-up evaluation were consistent with the initial outcomes.^8^ The patients who received surgery and achieved R0 resection had significantly improved OS and PFS. The 3-year OS rate for the R0 resection patients was 65.4%. The results showed that R0 resection was achieved for 53.2% of the patients and that 8.5% of the patients achieved pCR.

In addition, as shown in our previous report,^8^ induction therapy with the TPF regimen and subsequent conversion surgery was of reliable safety. No more than one third of the patients experienced grade 3 or 4 hematologic toxicity, and no serious perioperative complications or perioperative deaths were observed in the patients who received surgery.

Radical surgery remains the primary potentially curative treatment for resectable and borderline-resectable esophageal cancer, and R0 resection is of significance for the prognosis of BR-ESCC. Whether induction treatment is performed prominently affects the R0 resection rate, which can ultimately determine the prognosis of patients.^10–12^

Currently, only a few studies exist concerning the treatment strategy of BR-ESCC patients. A retrospective study performed by Suzuki et al.^12^ retrospectively analyzed 50 patients with BR-ESCC who underwent induction chemoradiotherapy (regimen: cisplatin and 5-fluorouracil plus 40-Gy irradiation). Of the 50 patients, 22 (44%) achieved R0 resection, and the median OS was significantly more favorable in the R0 group than in the non-R0 group (2.4 vs 0.8 years; HR, 0.28; 95% CI, 0.12–0.67; *p *< 0.01). However, for the patients in the study who failed to undergo R0 resection, the dose and field of induction radiation were insufficient, which increased the risk of disease progression. Furthermore, postoperative radiotherapy was not suitable for the patients who underwent R1 and R2 resection.

Another retrospective study^13^ identified the therapeutic effects of DCRT for BR-ESCC patients, with a 3-year OS of 46%. Nevertheless, the prognosis of DCRT is highly relevant to the tumor response. It is extremely difficult to perform salvage surgery routinely for esophageal cancer patients after DCRT due to the high risk of postoperative complications.

The COSMOS^11, 14^ study recruited 48 patients with BR-ESCC, and assessed the safety and efficacy of chemo-selection with docetaxel plus cisplatin and 5-fluorouracil (DCF) induction chemotherapy. The long-term follow-up results showed that the 3-year survival rate for all the patients was 46.6%, and that the prognosis of the patients who underwent R0 resection was significantly better than for those did not (3-year survival rate, 71.4% vs 30.1%). All the patients in our study received TPF chemotherapy as induction treatment, and the R0 resection patients also achieved better survival.

In summary, according to previous studies and the long-term follow-up results of the current study, induction therapy followed by R0 resection surgery can prolong long-term overall and PFS among BR-ESCC patients.

The NEOCRTEC-5010 trial^15, 16^ claimed that pCR was an independent positive prognostic factor for locally advanced esophageal cancer patients. In the current study, pCR was achieved in four patients (8.5%), which still was considerably lower than in the patients who received preoperative chemoradiation (pCR rate of NEOCRTEC-5010, 43.2%).^17^

The PLACE-1 trial^18^ confirmed that pembrolizumab combined with chemoradiotherapy (CRT) as preoperative treatment can further improve the pCR rate for patients with locally advanced squamous cell carcinoma (pCR rate of PLACE-1 trial, 55.6%).

In addition, ORIENT-15,^19^ another phase 3 trial, performed by Chinese researchers, showed that sintilimab combined with chemotherapy significantly improved the overall survival of patients with advanced esophageal squamous cell carcinoma compared with chemotherapy alone (median OS, 16.7 vs 12.5 months; *p*  < 0.0001; median OS of patients with CPS ≥ 10, 17.2 vs 13.6 months; *p *= 0.0018). Interestingly, all patients might benefit from sintilimab regardless of the programmed cell death ligand 1 (PD-L1) expression level. The tumor objective response rate (ORR) rate in the sintilimab group was 21% higher than in the control group, which was an impressive result and brought great confidence to advanced patients. Furthermore, sintilimab did not increase the adverse effects, which showed acceptable safety and tolerability. Thus, it is reasonable to consider that immunochemotherapy may improve the efficacy of BR-ESCC. As a result, our center is performing a phase 2 trial to explore whether induction immunochemotherapy (regimen: sintilimab, albumin-bound paclitaxel, cisplatin) can improve the R0 resection and pCR rates for BR-ESCC patients (ClinicalTrials.gov identifier: NCT04548440).

This study had some limitations. First, due to patient compliance, the treatment regimens (e.g., surgical methods and induction chemotherapy courses) were not implemented according to the plan. Second, the sample of this trial was small, and the calculation assumption of the sample size was based on the R0 resection rate of the previous 12 cases of surgery in our center alone. Therefore, the current study findings should be interpreted with caution.

In conclusion, long-term follow-up evaluation confirmed that TPF followed by surgery is feasible and promising in terms of survival for BR-ESCC patients. To further improve the R0 resection rate and prognosis, more effective induction treatment regimens need to be explored.

## References

[1] Sung H, Ferlay J, Siegel RL (2021). Global cancer statistics 2020: GLOBOCAN estimates of incidence and mortality worldwide for 36 cancers in 185 countries. CA Cancer J Clin.

[2] Abnet CC, Arnold M, Wei WQ (2018). Epidemiology of esophageal squamous cell carcinoma. Gastroenterology.

[3] Shah MA, Kennedy EB, Catenacci DV (2020). Treatment of locally advanced esophageal carcinoma: ASCO Guideline. J Clin Oncol.

[4] Lordick F, Mariette C, Haustermans K, Obermannová R, Arnold D (2016). Oesophageal cancer: ESMO clinical practice guidelines for diagnosis, treatment, and follow-up. Ann Oncol.

[5] Guideline of Chinese Society of Clinical Oncology (CSCO) (2021). Esophageal Cancer.

[6] National Comprehensive Cancer Network: NCCN Clinical Practice Guidelines in Oncology: Esophageal and Esophagogastric Junction Cancers, version 2, 2022. Retrieved xxxx at https://www.nccn.org/professionals/physician_gls/pdf/esophageal.pdf. 10.6004/jnccn.2019.0033 31319389

[7] Yokota T, Hatooka S, Ura T (2011). Docetaxel plus 5-fuorouracil and cisplatin (DCF) induction chemotherapy for locally advanced borderline-resectable T4 esophageal cancer. Anticancer Res..

[8] Wang Z, Hu M, Hu Y (2022). Paclitaxel plus cisplatin and 5-fluorouracil induction chemotherapy for locally advanced borderline-resectable esophageal squamous cell carcinoma: a phase II clinical trial. Esophagus..

[9] Shi CBJ, Branton PA, et al. (2017) Cancer protocol templates of the college of American pathologists (CAP). Protocol for the examination of specimens from patients with carcinoma of the esophagus. Version: Esophagus 4000.

[10] Nomura M, Kato K, Ando N (2017). Comparison between neoadjuvant chemotherapy followed by surgery and definitive chemoradiotherapy for overall survival in patients with clinical Stage II/III esophageal squamous cell carcinoma (JCOG1406-A). Jpn J Clin Oncol.

[11] Yokota T, Kato K, Hamamoto Y (2020). A 3-year overall survival update from a phase 2 study of chemoselection with DCF and subsequent conversion surgery for locally advanced unresectable esophageal cancer. Ann Surg Oncol.

[12] Suzuki T, Okamura A, Watanabe M (2020). Neoadjuvant chemoradiotherapy with cisplatin plus fluorouracil for borderline resectable esophageal squamous cell carcinoma. Ann Surg Oncol.

[13] Ishikura S, Kondo T, Murai T (2020). Definitive chemoradiotherapy for squamous cell carcinoma of the esophagus: outcomes for borderline-resectable disease. J Radiat Res..

[14] Yokota T, Kato K, Hamamoto Y (2016). Phase II study of chemoselection with docetaxel plus cisplatin and 5-fluorouracil induction chemotherapy and subsequent conversion surgery for locally advanced unresectable oesophageal cancer. Br J Cancer.

[15] Shen J, Kong M, Yang H (2021). Pathological complete response after neoadjuvant treatment determines survival in esophageal squamous cell carcinoma patients (NEOCRTEC5010). Ann Transl Med.

[16] Berger AC, Farma J, Scott WJ (2005). Complete response to neoadjuvant chemoradiotherapy in esophageal carcinoma is associated with significantly improved survival. J Clin Oncol.

[17] Yang H, Liu H, Chen Y (2018). Neoadjuvant chemoradiotherapy followed by surgery versus surgery alone for locally advanced squamous cell carcinoma of the esophagus (NEOCRTEC5010): a phase III multicenter, randomized, open-label clinical trial. J Clin Oncol.

[18] Chengqiang L, Shengguang Z, Yuyan Z (2021). Preoperative pembrolizumab combined with chemoradiotherapy for oesophageal squamous cell carcinoma (PALACE-1). Eur J Cancer.

[19] Lin Shen, Zhihao Lu, Junve Wang, et al. Sintilimab plus chemotherapy versus chemotherapy as first-line therapy in patients with advanced or metastatic esophageal squamous cell cancer: first results of the phase 3 ORIENT-15 study. 2021 ESMO LAB 52.

